# Third delay and associated factors among women who gave birth at public health facilities of Gurage zone, southern Ethiopia

**DOI:** 10.1186/s12905-023-02526-6

**Published:** 2023-07-12

**Authors:** Ayenew Berihun, Teshome Abuka Abebo, Bethelhem Mezgebe Aseffa, Yilkal Simachew, Meskerem Jisso, Yemisrach Shiferaw

**Affiliations:** 1 Gurage Zone Health Department, Welkite, Ethiopia; 2 School of Public Health, College of Medicine and Health Science, Hawassa University, Hawassa, Ethiopia

**Keywords:** Third Delay, Laboring mothers, Gurage Zone

## Abstract

**Background:**

The third delay is a delay in accessing emergency obstetric care timely and appropriately once a woman reaches a health facility. The third delay plays a crucial role as an indicator to assess the quality of obstetrics services and is often the leading contributing factor to maternal mortality in developing countries. Although considerable research has been conducted on pre-facility delays in healthcare access, there is a lack of focus on delays experienced upon arrival at health facilities, particularly in Ethiopia and the specific study areas of Gurage zone. This study aimed to assess the magnitude of the third delay and associated factors among women who gave birth at Public Health Facilities of Gurage Zone, Southern Ethiopia.

**Method:**

A facility-based cross-sectional study was conducted with 558 women who gave birth at public health facilities of Gurage Zone from January 01/2020 to March 30/2020. Multi-stage stratified sampling technique was used to select the nine facilities. The data was collected using a structured interviewer administer questionnaire and an observational checklist. Women who waited more than an hour to receive delivery services after arriving at the health facility were classified as experiencing the third delay. The data were entered and analyzed using Epi Data version 3.1 and SPSS version 20.0 software, respectively. Binary logistic regression was employed to identify the determinant factors for the third delay. Variables having a P-value < 0.25 in the binary analysis were a candidate for multivariable analysis. Variables with P < 0.05 were considered statistically significant.

**Result:**

The magnitude of the third delay was 193 [(34.8%; 95% CI; (30.8%, 38.8%)]. Complication during labor [AOR = 2.0; 95% CI, (1.4, 3.0)], Presence of functional generator in a health facility [AOR = 2.8; 95% CI, (1.3, 6.3)], level of health institution [AOR = 2.8; 95% CI, (1.04, 7.8)] and BEMONC training in the last two years [AOR = 1.6; 95% CI, (2.0, 6.5)] were significantly associated with third delay.

**Conclusion:**

The magnitude of third delay was high compared to some low income countries, which shows most of mothers were not getting the service timely after they arrived at the health facility. Equipping health facilities with trained manpower and with necessary materials and infrastructure will contribute to hastening the provision of obstetric care.

**Supplementary Information:**

The online version contains supplementary material available at 10.1186/s12905-023-02526-6.

## Introduction

The current global maternal mortality ratio (MMR) (211/100,000 live birth) is unacceptably high, and the vast majority, 94% of these deaths occur in low and middle-income countries [1]. Ethiopia is among the 16 Sub-Saharan countries with the highest MMR of 412 per 100,000 live births. The differences in MMR between low and high-income countries arise from differences in healthcare service utilization and in the quality of care provided. Evidences shows that hemorrhage, hypertensive disorders, abortion, and sepsis are the primary causes of maternal death. More than 80% of these deaths can be prevented through the timely provision of existing emergency obstetric interventions [2].

The three delays increase the risk of maternal death because of the delay in deciding to seek health care (first delay), delay reaching a health facility (second delay), and delay in receiving medical care timely and appropriately once reached in a health facility (third delay) [3]. The first two delays are related to demand-side barriers that prevent women from utilizing and accessing delivery services [4]. The third delay in accessing maternal health care is closely linked to various factors associated with health facilities and the quality of care provided. These factors encompass the absence of emergency obstetric care services and supplies, shortage of trained staff, poor management of emergency obstetric care provision, long waiting times, poor referral practices, and poor coordination among staff [5, 6].

In low-income countries, a considerable number of mothers died or faced adverse maternal outcomes after they had reached healthcare facilities [7–10]. Therefore on top of ensuring skilled birth attendant assisted deliveries, timely provision of quality obstetric care is needed to reduce maternal mortality. However, various studies in Ethiopia have revealed that more than one-third of mothers who visited health facilities for obstetric care experienced a third delay [11–13].

Timely access to Emergency obstetric and newborn care (EmONC) services is one of the most essential strategies for reducing maternal and neonatal mortality in low-income countries like Ethiopia. It is an evidence-based service required to manage potentially life-threatening complications affecting many women and newborns during pregnancy, childbirth, and the immediate postpartum period [14]. Appropriate implementation of EmONC can significantly reduce maternal and perinatal death and complications [15–17]. Despite this, EmONC services are not widely available and accessible to women in Ethiopia due to poor infrastructure, insufficient resources, and a lack of manpower [18].

A third maternal delay is an indicator of poor quality of obstetric care [19]. Despite, there has been significant research conducted on delays prior to reaching health facilities, limited attention has been given to the delays experienced upon arrival at the health facility in Ethiopia and the specific study areas of Gurage zone. Understanding the factors that contribute to third delays could lead to improvements in the quality of obstetrics service [16]. Therefore, this study aimed to assess the magnitude of the third delay and associated factors among women who gave birth at public health facilities in the Gurage zone.

## Methods

### Study design and setting

A facility-based cross-sectional study was conducted among laboring mothers attending public health facilities in Gurage Zone Southern Ethiopia from January 01/2020 to March 30/2020. Gurage Zone is located 160 km South West of Addis Ababa, the capital city of Ethiopia and 240 km from Hawassa, the capital city of South Ethiopia. Welkite is the administrative center of the Zone. According to the 2011 Ethiopian population census, the zone’s total population would be 1,867,377 in 2020. From this population 952,363 were females, and 915,014 were males. Gurage Zone is divided into 16 woredas and four administrative cities, with 412 rural and 32 urban kebeles. There are 5 Public Hospitals, 65 Public Health centers, 444 Health Posts, 8 Private Health centers and 2 Private Hospitals.

### Study population

All women who give birth at public health facilities of Gurage zone were the source population. All women who gave birth at the selected public health facilities of Gurage zone during the study period were the study population. Women who were critically ill and unable to communicate during the data collection period were excluded from the study.

### Sample size

#### Sample size determination for specific objective 1

The sample size was determined using the formula for single population proportion by considering the third delay proportion of 32.6% from a study conducted in Hadiya Zone, Southern Ethiopia [11] with a 95% level of confidence, 5% margin of error to be tolerated and 10% non-response rate added.

$$n=\frac{{Z}^{2 }p(1-P)}{{d}^{2}}$$

Where: d = margin of error (5% = 0.05).

P = best estimate of population proportion (32.6% = 0.326).

q = 1‒P = 0.674.

Z = confidence level (95% = 1.96).

n = sample size.

$$n = \frac{{{z^2}p{\text{ }}q}}{{{d^2}}} = \frac{{{{\left( {1.96} \right)}^2}\left( {0.326} \right){\text{ }}\left( {0.674} \right)}}{{{{\left( {0.05} \right)}^2}}} = 338$$

After considering a 10% non-response rate and 1.5 design effects, the final sample size was 558.

### Sample size determination for specific objective 2

The sample size calculation for the second objective, which is to identify the determinants of the third delay, was calculated using the double population proportion formula by using Epi info version 7 stat calc programs (Table 1).

**Table 1:** Sample size calculation for the second Objective to identify associated factors of the third delay by using Epi info software

Variables	CI (%)	Power	Ratio	% of outcome among exposed	AOR	Sample size (n)
Multiple referral level [20]	95	80%	1:1	21.6	2	260
Uncooperative staff [21]	95	80%	1:1	21.1	2	256
Absence of care provider [20]	95	80%	1:1	59.7	2	370

### Sampling procedures

**Fig. 1:**
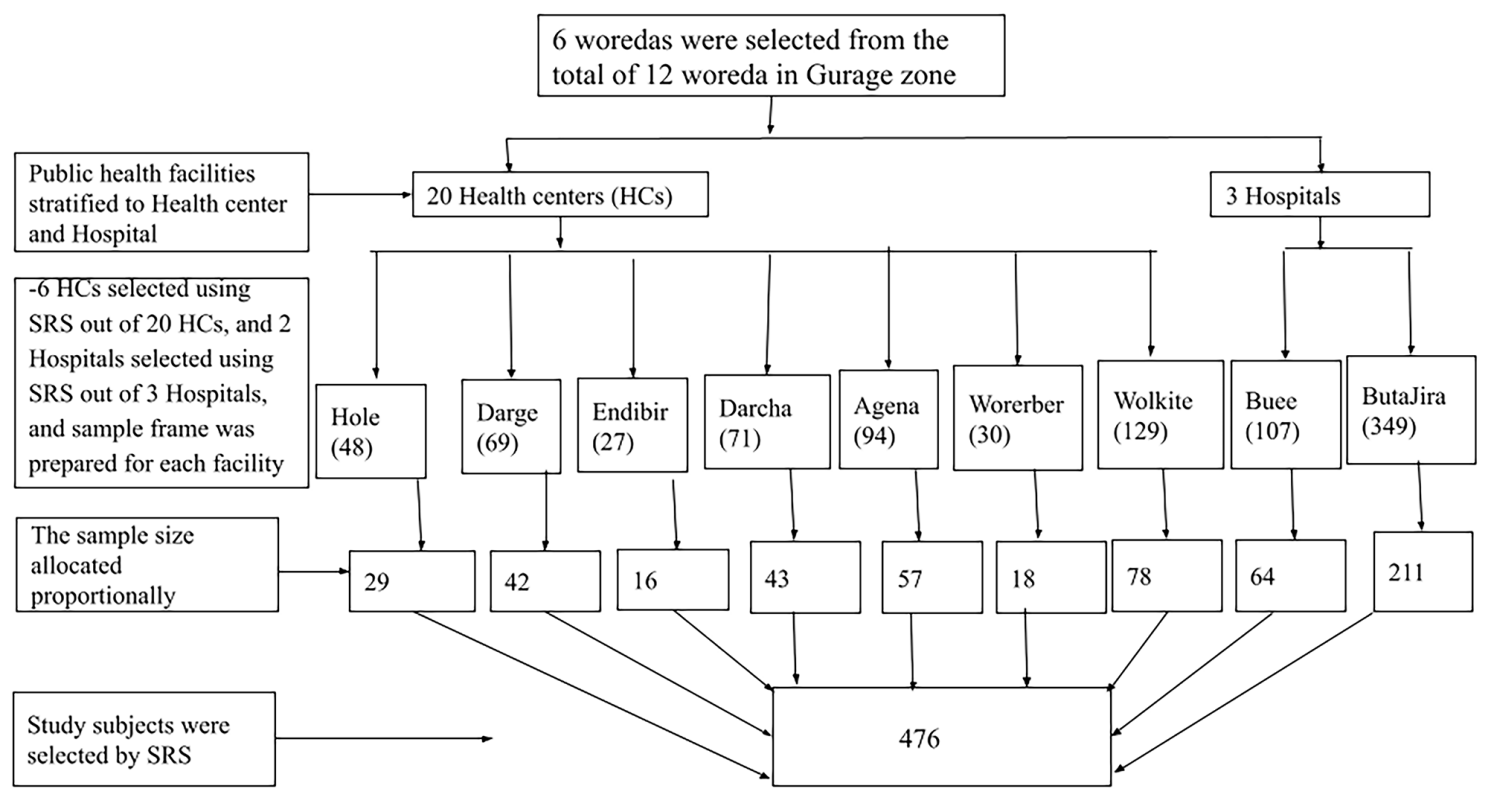
Diagrammatic presentation of sampling procedure

First, we randomly selected six woredas out of the total 16 woredas in the Gurage zone. Within these selected six woredas, the public health facilities were stratified into hospitals and health center. Then, a total of seven health centers were randomly selected from a pool of twenty health centers, while two hospitals were randomly chosen from three hospitals. The total sample size was proportionally allocated to selected health facilities based on their previous three months’ delivery load. Then, from January 1 to March 30/2020, study participants were selected and interviewed in each health facility using a simple random sampling technique (Fig. 1).

### Variables of the study

Dependent variable: Third maternal delay.

Independent variables: Socio-demographic and economic factors, Health-care system related factors.

#### Operational definition

Third maternal delay: the time between arriving at the facility and receiving the delivery care service. Time taken ≥ 1 h to receive delivery service considered a delay and less than an hour is considered no delay [22].

### Data collection instruments and method

The data was collected using a structured interviewer administer questionnaire and an observational checklist. The questionnaire was adapted from JHPIEGO tools [23] and indicators for maternal and neonatal health and relevant literature sources [10, 11, 24, 25]. The tool contains the following sections; socio-demographic characteristics, third delay and information during arrival, and health care system related factors. The socio-demographic characteristics and information about the laboring mother’s arrival at the health facility were gathered using the interview-administered questionnaire. An observational checklist was used to assess the third delay by measuring the time elapsed from a mother’s arrival at a health facility to when she received obstetrics service. Additionally, an observational checklist was used to assess healthcare system factors like human resources, availability of medical supplies, drugs, and infrastructure.

### Data quality control

The questionnaire was prepared in English and translated to the local language, Amharic and Guragigna, and then back to English to ensure consistency. A pretest was done on 5% of the sample size before the actual data collection. A necessary amendment was made following the result of pre-test. The investigators trained data collectors and supervisors about the study objectives, data collection tools and procedures, confidentiality, and informed consent for two days. Ten Obstetric nurses fluent in Amharic and Guragigna were recruited for data collection. During data collection, the supervisors closely supervised, checked, and reviewed the data daily for clarity, consistency, and completeness.

### Data processing and analysis

Data were entered into EpiData version 3.1 and exported to SPSS version 20.0 for analysis. Descriptive statistics were computed and results were presented using table and narration. Both binary and multivariable logistic regression analysis were carried out to identify factors associated with the third delay. Variables with P-value < 0.25 in binary logistic regression were considered candidates for the final model. In the multivariable logistic regression, a significance level was declared at p < 0.05, and an adjusted odds ratio (AOR) with a 95% confidence interval (CI) was used to measure the association. The variance inflation factor (VIF < 10) was used to check the multi-collinearity between candidate variables. The model fitness was checked by Hosmer and Lemeshow goodness of fit test.

## Results

### Socio-demographic characteristics of the respondents

A total of 555 respondents were involved in the study yielding a 99.5% response rate. Of these, 289(52.1%) were rural dwellers, and the mean (± SD) age of the study participants was 28.2 (± 5.5). More than half of the respondents, 280 (50.5%), were Orthodox Christian in religion. The dominant ethnicity was Gurage, 423 (76.2%). Regarding educational status, 156 (28.1%) cannot read and write. Nearly 530 (95.5%) mothers were married, and 330(59.9%) of the study participants were housewives. The study participants’ mean (± SD) monthly household income was 2,079.3(± 1484.3). More than half of the mothers 304(54.8%) were members of community-based health insurance schemes **(**Table 2**).**

**Table 2:** Socio-demographic characteristics of laboring mothers in Gurage Zone, Southern Ethiopia, March 2020 (n = 555)

Variables	Frequency (%)
Residence	
Urban	266 (47.9%)
Rural	289 (52.1%)
Religion	
Orthodox	280 (50.5%)
Muslim	182 (32.8%)
Protestant	78 (14.1%)
Catholic	15 (2.7%)
Ethnicity	
Gurage	423 (76.2%)
Amhara	49 (8.8%)
Oromo	32 (5.8%)
Wolaita	24 (4.3%)
Others	27 (4.9%)
Marital Status	
Married	530 (95.5%)
Other	25 (4.5%)
Education of mothers	
Formal education	314(56.6%)
Non formal education	241 (43.4%)
Occupation of mother	
Housewife	330 (59.5%)
Government Employed	77 (13.9%)
Merchant	94 (16.9%)
Non-government employed	52 (9.4%)
Others	2 (0.4%)
CBHI	
Yes	304 (56.8%)
No	251 (45.2%)

CBHI = Community-based health insurance

### Health facility characteristics

#### Infrastructure

The data was collected from nine health facilities (7 health centers and 2 Hospitals). Of them, six health facilities (4 health centers and one hospital) did not have any functional telephone. Functional generator was found in 6 health facilities (2 hospitals and 4 health centers). Two hospitals and five health centers had functional ambulances or other vehicles. The total number of beds across all facilities was 243, with 53 designated for delivery service.

### Obstetric and newborn care services

The following services were available in all health facilities; normal and assisted vaginal delivery service, parenteral administration (IV/IM) of antibiotics, oxytocin, and anti-convulsion. Blood transfusion service was available only in one hospital, and caesarean section service was provided in the two hospitals. Of all health facilities, 2 hospitals and 4 health centers had the national guidelines for integrated management of pregnancy and childbirth (IMPAC). In the last two years, delivery service providers in seven health facilities (1 hospital and 6 health centers) have received training on BEMONC.

### Essential medicines

In terms of medication availability, all study health facilities had Diazepam, Magnesium Sulphate, and Gentamycin injection. Calcium gluconate injection and Ampicillin Powder for injection were available in 6 and 7 of the health facilities, respectively.

### Delivery services and the ratio of health providers

In terms of work time, 40 health providers work during the day, and the number of deliveries encountered during the day was 23[maximum 8 in hospitals and minimum 2 in health centers], and the ratio was 1.76. Twenty-two health providers work at night time, the number of delivery faced at night time was 47 [maximum of four in hospitals and a minimum of two in health centers] and the ratio was 2.6 **(**Table 3**).**

**Table 3:** Delivery services and the ratio of health providers in the study health facilities in Gurage zone, Southern Ethiopia, March 2020 (n = 9)

Variables	Min	Max	Mean	SD
No. of health providers during the day	2	8	4.4	1.74
No. of health providers during the night	2	4	2.44	0.73
No. of delivery service during the day	1	7	2.6	2.2
No. of delivery service during the night	1	24	5.2	7.5

SD, Standard Deviation

### Magnitude of third delay and information on arrival

Majority of the respondents 448(80.7%) had labor during their arrival. More than one-third 193 [(34.8%; 95% CI; (30.8, 38.8)] of the study participants were waiting for more than an hour to receive definitive obstetric care. Hospital delays accounted for 140 (72.5%) of all cases. The mean delay time was 1.45 h. Among the participants with delayed care 106 (54.9%) had complication. Of the delayed 22 (11.4%) were referred from other health facilities. More than half 313 (56.4%) of the respondents arrived during the day time. Weekends and holidays deliveries constitute nearly a quarter 133 (24%) of the whole deliveries **(**Table 4**).**

**Table 4:** Timeliness and arrival information of laboring mothers in Gurage Zone, Southern Ethiopia, March 2020(n = 555)

Variables	Frequency (%)
Arrival in labor	
Yes	448 (80.7%)
No	107 (19.3%)
Waiting time to receive obstetric care	
≥1 h	193 (34.8%)
≤1 h	362 (65.2%)
Time of arrival	
Day	313 (56.4%)
Night	242 (43.6%)
Date of arrival	
Work day	422 (76%)
Weekend/Holiday	133 (24%)
Complication was diagnosed	
Yes	210 (37.8%)
No	345 (62.2%)
Referral case	
Yes	54 (9.7%)
No	501 (90.3%)
Level of health facility	
Health center	283 (51%)
Hospital	272 (49%)

### Factors associated with the third delay

During the univariate analysis, the presence of complications, availability of generator, availability of retained placenta removal service, level of the facility, presence of IMPAC, training on BEMOC for delivery service providers, number of workers during the day, availability of Calcium gluconate injection, Sodium chloride injectable and Azithromycin were found to be significantly associated with the third delay at P-value ≤ 0.25. In multivariable logistic regression analysis, the presence of complication, the availability of functional generator, the level of facility and the status of BEMONC training in the last two years were considered factors affecting the third delay in Gurage Zone.

The odds of delay in receiving obstetrics care among mothers with complication during labor were 2 times higher than mothers with no complication [AOR = 2.0; 95% CI, (1.4, 3.0)]. Third delay was 2.8 times higher among mothers who gave birth in health facilities without functional generator [AOR = 2.8; 95% CI, (1.3, 6.3)]. Maternal delay after arriving at the health facility was 2.8 times higher among mothers who delivered in a hospital than a health center [AOR = 2.8; 95% CI, (1.04, 7.8)]. The risk of delaying in receiving obstetric care was 3.6 times higher among mothers who attended health facilities where delivery attendants did not receive BEMOC training in the last 2 years [AOR = 3.6; 95% CI, (2, 6.5)] **(**Table 5**).**

**Table 5:** Multivariable logistic regression analysis of factors associated with third delay among laboring mothers in Gurage Zone, Southern Ethiopia, March 2020 (n = 555)

Variables	Third delay		COR(95% CI)	AOR( 95% CI)
	**Delayed**	**Not Delayed**		
Complication was diagnosed Yes No	106(54.9%) 87(45.1%)	104(28.7) 258(71.3%)	3(2.1, 4.4) 1	2(1.4, 3.0) * 1
Availability of functional generator Yes No	173(89.6%) 20(10.4%)	295(81.5%) 67(18.5%)	1 0.5(0.3, 0.87)	1 2.8(1.3, 6.3)*
Level of health facility Hospital Health Center	140(72.5%) 53(27.5%)	132(36.5%) 230(63.5%)	4.6(3.1, 6.7) 1	2.8(1.04, 7.8)* 1
Presence IMPAC Yes No	169(87.6%) 24(12.4%)	272(75.1%) 90(24.9%)	2.3(1.4, 3.8) 1	1.3(0.42, 4.2) 1
Training on BEMOC within 2 years Yes No	59(30.6%) 134(69.4%)	242(66.9%) 120(33.1%)	1 4.6(3.1, 6.7)	1 3.6(2.0, 6.5)*
Availability of Calcium gluconate injection Yes No	170(88.1%) 23(11.9%)	273(75.4%) 89(24.6%)	1 0.42(0.25, 0.68)	1 1.9(0.7, 5.1)

*Significant at p value < 0.05, C/S = Caesarean Section, BEMOC = Basic Emergency Obstetrics Care, IMPAC = national guidelines for integrated management of pregnancy and child birth

## Discussion

The finding of this study showed that magnitude of third delay was 34.8%. The presence of complications, the availability of functional generator, the level of facility and the status of BEMONC training in the last two years were found to be significantly associated with the third delay.

This study showed that the magnitude of the third delay was 34.8% which was comparable with the findings in Bahir Dar (30.7%), Yem (34.7%), tertiary hospital in Southern Ethiopia 32.3%, and Gamo Zone (31.7%) [11, 22, 24, 26]. But it was higher than finding in Mozambique (14.2%) [8]. The discrepancy could be explained by the fact that the study in Mozambique used secondary data and solely measured delay that occurred in maternal death; those mothers who survived but had faced a delay in receiving maternal care were not included. On the contrary, the magnitude of the current finding was by far lower than the findings in Addis Ababa (74.7%) and Malawi (96.8%), respectively [9, 13]. Methodological differences might be the reason for the discrepancy. The study in Addis Ababa was done in two tertiary referral hospitals where complicated cases are referred. Therefore, the nature of the cases and the additional time required for the referral procedure could cause the third delay. The study in Malawi was conducted in one of its districts by including all maternal deaths (that occurred in community and health care facilities) during one year. The other reason could be the difference in the definition of third delay; in Malawi’s study, it was defined as “waiting for more than 30 minutes”.

The mean delay time of this study was 1.45 h, which was comparable with the mean delay time in the Gamo zone 1.56 h but it was lower than the mean delay time (2 h) in Hossaina; and Bahir Dar 4 h [11]. The difference could be due to a variation in the study environment and a difference in assessing delay. The study in Hosaena was done in one hospital but, the current study consists of hospitals and health centers. The source of information for the time interval after reaching health facilities to receiving obstetric care in Bahir Dar study was the study participants, and more over the current study was conducted seven years later than Bahir Dar’s study, so the time difference may attributed to the difference.

In this study third delay was higher among mother who had complication, this finding is supported by the finding conducted in Bahir Dar, Ethiopia [24]. These could be explained by, the presence of complications might necessitate additional diagnostic procedures or consultations with specialists, which can contribute to longer wait times for receiving care.

For effective emergency and obstetric care, there should be an “enabling environment” which includes sufficient human and financial resources, essential drugs, and the necessary equipment [27]. According to this study, the magnitude of the third delay was higher in health facilities that didn’t have a functional generator. This was supported by findings in two systemic review conducted in low and middle-income countries [28, 29]. This implies that when a healthcare facility lacks a reliable power source, there are increased delays in providing appropriate care to women in need. Without functional generators, healthcare providers may face challenges in performing essential procedures and interventions, which can lead to delays in providing appropriate care during critical moments.

The finding of the current study indicates a higher likelihood of experiencing the third delay among mothers who received delivery services from hospitals compared to those who utilized health centers. This suggested that mothers accessing delivery services at hospitals encountered more delays in receiving timely and appropriate care upon arrival. This finding was supported by studies conducted in Mozambique and Malawi [8, 9]. This might be because of the high staff workload, and patient flows in the hospital. In Malawi’s study, long waiting time was mentioned as a possible reason for the higher third delay in hospitals than in health centers [9]. To reduce the third delay, qualified services providers in the right quantity must be combined with the availability of supplies for emergency care (drugs, blood) and the structural functioning of the health facilities, such as the functionality of the operating room [30]. However, this study showed even though both hospitals provide caesarian section services, Blood transfusion service was available only in one hospital. Blood transfusion is one of the signal functions used to manage serious obstetric complications such as bleeding, ectopic pregnancy, and uterine rupture. The absence of this service would cause the emergency obstetrics response to be delayed.

The study revealed that mothers who get the delivery service from health providers without BEMONC training were more likely to experience a third delay than mothers who get the service from BEMONC trained health providers. This showed providing Basic emergency obstetric and neonatal care (BEMONC) training for delivery service providers will reduce the magnitude of the third delay. This finding is in line with studies conducted in Gamo zone and Hadiya [11, 26]. It has been mentioned in different literature that providing BEMONC and other training will improve the performance of obstetric care providers so as it will shorten the delays in diagnosis, decision and referral [29]. This could be explained, by BEmONC training for health center practitioners at the primary health care unit can potentially alleviate the case flow burden on hospitals. By equipping health professionals at the primary health care units with the necessary skills and knowledge in basic emergency obstetric and newborn care, they can effectively manage and handle uncomplicated deliveries. This empowers health centers to take on a large share of low risk-births, consequently reducing the number of such cases being referred to hospitals. As a result, hospitals can prioritize their resources and expertise for more complex and high-risk cases, ensuring better allocation of healthcare resources and optimizing overall care. In addition, since BEmONC was not a standard part of pre-service training for all maternal healthcare providers, the absence of in-service BEmONC training might leave the healthcare facilities unprepared to identify and respond to obstetric emergencies The contribution of unskilled health professional for the third delay and maternal death at a health facility is not negligible [31–33].

### Strength and limitation of the study

The study was conducted promptly after the women received delivery service, which greatly reduces the potential for recall bias. Additionally, the time taken to receive obstetric care was directly observed by the data collectors and cross-checked with the information provided by the mothers, thereby enhancing the accuracy of the study.

The study excludes private health institutions, so the findings are intended to be applicable to only public health institutions. Even if the study tried to examine the institutional and individual factors of maternal third delay, causal relationship can’t be established due to the nature of the study design. Additionally, since the interviews were conducted within the institution, there is a possibility that mothers may have felt restricted in providing negative feedback regarding the service they received.

## Conclusion

The magnitude of the third delay was high, which shows most mothers were not getting the service timely arriving at the health facility. Presence of complications, availability of functional generator, level of facility and status of BEMONC training in the last two years were factors associated with the third delay. Therefore, emphasis should be given to equipping health facilities with the necessary resources like functional generator and improving the capacity of obstetric care providers with BEMONC training. We recommend future researchers to investigate the effect of the third delay on the pregnancy outcome.

## Electronic supplementary material

Below is the link to the electronic supplementary material.

Supplementary Material 1

## Data Availability

All data generated or analyzed during this study are included in this article.

## Abbreviations

ANC: Antenatal care
BEMOC: Basic emergency obstetric care
CBHI: Community-based health insurance
EMOC: Emergency obstetrics care
EMONC: Emergency obstetrics and neonatal care
MMR: Maternal mortality rate
SDG: Sustainable development goals
SNNPR: Southern nations, nationalities, and people’s region
TBA: Traditional birth attendant
